# Ecological recognition of self-esteem leveraged by video-based gait

**DOI:** 10.3389/fpsyt.2022.1027445

**Published:** 2022-10-10

**Authors:** Xingyun Liu, Yeye Wen, Tingshao Zhu

**Affiliations:** ^1^Key Laboratory of Adolescent Cyberpsychology and Behavior, Ministry of Education, School of Psychology, Central China Normal University, Wuhan, China; ^2^CAS Key Laboratory of Behavioral Science, Institute of Psychology, Chinese Academy of Sciences, Beijing, China; ^3^Department of Psychology, University of Chinese Academy of Sciences, Beijing, China; ^4^School of Electronic, Electrical, and Communication Engineering, University of Chinese Academy of Sciences, Beijing, China

**Keywords:** self-esteem, gait pattern, machine learning, video, 2D

## Abstract

Self-esteem is a significant kind of psychological resource, and behavioral self-esteem assessments are rare currently. Using ordinary cameras to capture one’s gait pattern to reveal people’s self-esteem meets the requirement for real-time population-based assessment. A total of 152 healthy students who had no walking issues were recruited as participants. The self-esteem scores and gait data were obtained using a standard 2D camera and the Rosenberg Self-Esteem Scale (RSES). After data preprocessing, dynamic gait features were extracted for training machine learning models that predicted self-esteem scores based on the data. For self-esteem prediction, the best results were achieved by Gaussian processes and linear regression, with a correlation of 0.51 (*p* < 0.001), 0.52 (*p* < 0.001), 0.46 (*p* < 0.001) for all participants, males, and females, respectively. Moreover, the highest reliability was 0.92 which was achieved by RBF-support vector regression. Gait acquired by a 2D camera can predict one’s self-esteem quite well. This innovative approach is a good supplement to the existing methods in ecological recognition of self-esteem leveraged by video-based gait.

## Introduction

The research history of self-esteem is almost as long as the research history of scientific psychology itself (1). Self-esteem is defined as one self’s evaluation of, or attitude toward, him-or herself initially (1). The research in this area has continually improved over time. Scholars’ enthusiasm hasn’t lessened, either. Many researchers have come to an agreement that self-esteem could provide a buffer or serve as a coping strategy against negative experiences such as anxiety (2), negative feedback (3), psychological distress (4), suicide (5), and so on. At the same time, the bulk of research demonstrates that self-esteem has positive benefits for people’s health (6), achievement (7), relationship quality (8), well-being (9), and so on, especially for students (10).

Self-esteem is so crucial that numerous methods to measure it have been presented. Currently, questionnaires have an edge in the field of self-esteem assessment, especially self-report ones (7). Rosenberg Self-Esteem Scale (RSES), for example, is one of the most frequently utilized surveys (11). For the last three decades, researchers also paid attention to the measurement of implicit self-esteem, such as the Implicit Association Test (IAT) and Name–Letter Test (NLT) (12). Aside from self-reporting, behavioral evaluation is offered to measure self-esteem with the assistance of computer technology. Gait is the dynamic change in the human body during walking which serves as a motion representation of body posture. Gait can manifest individuals’ health status, emotions, personality, and self-esteem (12, 13). For example, Sun and her colleagues used Kinect to collect participants’ gait data. Then they predicted participants’ self-esteem levels by machine learning model. Their results showed that the best correlation coefficient between predicting models and self-report scores for self-esteem was 0.45, which exhibited the validity of this newly developed method (14).

Because each method has its own set of advantages and disadvantages, arbitrarily deciding which method is superior is both impracticable and pointless. Meanwhile, a variety of resources and methodologies may help the advancement of evaluation (15). Even while behavioral evaluation is a useful supplement to questionnaires for participants who cannot read or write, such as the young, old, crippled, and illiterate, the growth of these two domains is not synchronous. To the best of our knowledge, only sporadic research carried out to testify the validation of behavioral assessment. In addition to the research mentioned above (14), Wang and his colleagues employed Kinect to record participants’ facial expressions during self-introduction to predict participants’ self-esteem using machine learning models (16). The two studies share the same issue in that all of their findings are based on 3D data captured by the Kinect, however, in the real world outside of laboratories, the behavior recording instruments are generally 2D such as the surveillance equipment or video cameras in a public place. This severely restricts the capacity of current technology to support scientific research or urban governance. Meanwhile, current studies lacked a complete examination of the model’s performance, leading to uncertainty about the model’s reliability.

Nowadays, some researchers start to pay attention to 2D data to measure personality. For instance, Yeye and his colleagues used a 2D camera to predict personality by machine learning models (17). Due to the significance of self-esteem and the prevalence of 2D cameras in modern society, we used an ordinary 2D video camera to record gait data for this study. We then trained machine learning models to measure self-esteem automatically to optimize how this study can be put to use in practice with the reliability and validity of the reached standards.

## Materials and methods

### Participants

Posters on campus were used to find postgraduate students at a large north China university who were older than eighteen and did not have any physical impairment that would have made it difficult for them to walk. The experiment included 153 participants in all, and each received 100 RMB as payment for their participation. All of the participants signed written informed consent forms before the formal experiment. Only one participant was disqualified due to missing data. Finally, there were 152 participants’ data analyzed in this study (79 males and 73 females; mean age = 23.00°years, SD = 1.07°years). The study was approved by the Institutional Review Board of the Institute of Psychology at the Chinese Academy of Sciences (approval number: H15010).

### Materials and instruments

**Rosenberg Self-Esteem Scale (RSES)** (11) was used in this experiment. Participants indicated the extent to which they agreed with each of the 10 items on the RSES. Sample items include, “I feel that I am a person of worth, at least on an equal plane with others.” and “I feel that I have a number of good qualities.” (1 = Strongly disagree, 4 = Strongly agree). A higher score on this scale indicates higher self-esteem. The Chinese version of the RSES showed good validity and reliability with the Cronbach’s α coefficient is 0.83 (18).

**FIGURE 1:**
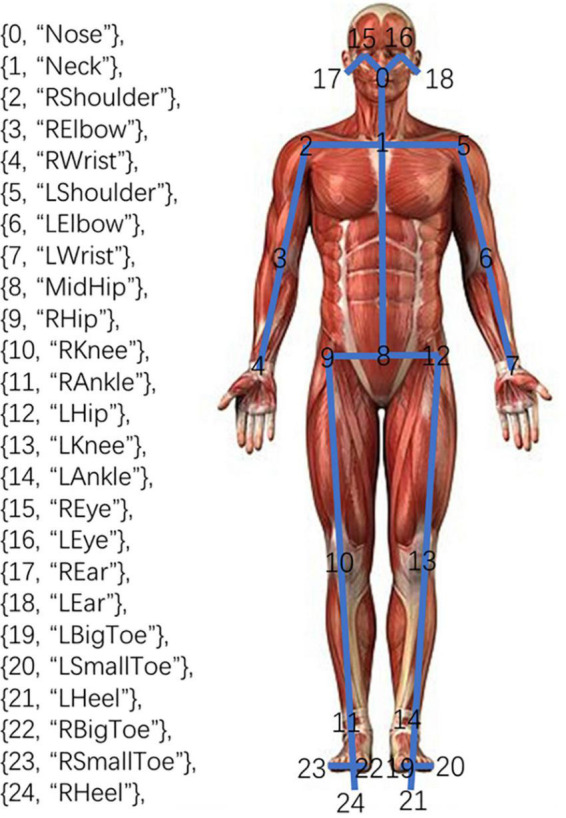
The 25 joints captured by the BODY25 model.

**OpenPose human posture recognition system** was employed in our study. The system is an open-source project launched by Carnegie Mellon University. It can detect key points of the body trunk, face, fingers, and toes. The BODY25 model, the subsystem of body posture detection in OpenPose, can realize the two-dimensional coordinate detection of the 25 key points of the body trunk, that is, the real-time output of the two-dimensional coordinates of each key point of the body trunk to be detected, and the confidence of the coordinates of the point (19). The key points were shown in Figure 1, including the Nose, Neck, and other key features.

### Procedure

**FIGURE 2:**
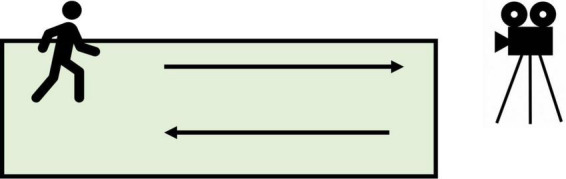
The experiment procedure of the study.

In a noiseless room, a rectangular carpet (6 m × 2 m) was laid up. The position of the video camera with a 25 Hz sampling rate was determined according to the position of the rectangular carpet and the light, which ensured that the whole body of the participants can be photographed continuously without obstruction. The participants were first required to walk back and forth on the rectangular carpet as they typically did until they heard the stop command, which lasted for 2°min (as shown in Figure 2). Then participants completed the RSES and some other questionnaires so that they could figure out the purpose of the research.

### Data preprocessing

First, there were face-toward and back-toward parts in relation to where the camera was pointed. Because the face-toward segments’ accuracy was higher than that of the back-toward segments (20), we chose them for feature extraction. We just needed to extract a cycle of data for analysis because gait is a cyclic physical activity. Following data segmentation, a complete gait segment for each participant was chosen, which included the face-toward gait segments from far and near as seen by the video camera.

Second, we eliminated the differences in the relative positions of the participants with the video camera and the shape diversity by using the SpineBase joint, i.e., No. 8, the MidHip, as the reference point in every frame to adjust the 2D coordinates of the various participants into the same coordinate system. In particular, the MidHip’s 2D coordinates were subtracted from each joint’s original coordinates to determine its new 2D coordinates. We used the additional 24 joints to extract features.

**FIGURE 3:**
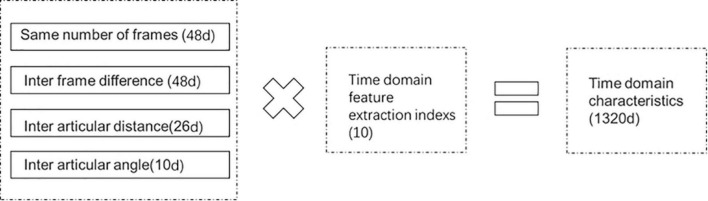
Illustration of time characteristic dimension.

Thirdly, due to the accuracy of the OpenPose system and the background interference of gait video, the key point coordinates often have high-frequency noise interference in the process of key point coordinate extraction (Figure 3 left). Therefore, we ran a Gaussian filter, i.e., a low-pass filter (21) on the data for every dimension (X and Y) of the 25 joints to remove noisy data. The window length was five, and the convolution kernel of the Gaussian filter was *c* = [1, 4, 6, 4, 1]/16. The "In" represented the initial data that the video camera had recorded, while the "Out" represented the fresh data. The equation read as follows:


(1) $$O\,u\,t\,\left[i\right]=\frac{1}{16}\,\left(I\,n\,\left[i\right]\times 1+I\,n\,\left[i+1\right]\times 4+I\,n\,\left[i+2\right]\times 6+I\,n\,\left[i+3\right]\times 4+I\,n\,\left[i+4\right]\times 1\right)$$

Finally, after the data segmentation process, the number of frames in the retained gait segments varied due to the varying stride lengths and speeds of each participant. Each participant kept at least four full gait cycles to prevent the impact of different gait data on modeling and to account for the integrity of gait. In this study, 75 gait frames were used to represent all of the individuals. Due to the 25 frames per second frame rate of the video, each participant’s gait was recorded for 3°s as a single segment.

### Feature extraction and selection

#### Time-domain features

The modifications of each frame contain the movement information of gait, therefore rather than studying the human posture in each frame independently, we should combine them and concentrate on the changes between them. The inter-frame difference method was used in this work, and the key point coordinates of two adjacent frames were used for various procedures that indicated the motion properties of the gait (i.e., Δ*f*_*k*_ = *f*_*k* + 1_ − *f*_*k*_).

The limbs shift in conjunction with one another during gait and comply with the fundamental biological kinematics principle (22, 23). Gait studies should therefore pay attention to the interaction and change trend of joints in gait rather than only concentrating on the motion law of a key point or a joint in isolation. In a previous study, the angle between joints served as a crucial metric to assess the movement trend between joints (24). This study used the angle between joints to describe the changes between numerous joints in a local motion system made up of several joints, such as the shoulder, hand, and wrist joints that make up the bending motion of the arm. In biological movement, the angle between joints also has a corresponding meaning. For instance, the angle between key points No. 0, 1, and 2 denotes the tilt of the human head at this precise moment; the angle between key points No. 9, 10, and 11 indicates the current bending motion of the right knee of the human body. Ten joint angle indicators in total were suggested for this study (Table 1). Particularly, A0 and A1 stand in for the neck’s right and left tilt angles, respectively. The two are not redundant since the individuals’ shoulders may have shrugged, been high or low, or have other variations throughout gait movement, preventing the left and right angles of their necks from forming a flat angle. Meanwhile, we utilized angle 12_9_10 and angle 9_12_13 for A6 and A7 instead of and angle 8_9_10 and angle 8_12_13, respectively, because N0. 8 was removed during data preprocessing.

**TABLE 1:** The meaning of angle between joints.

Number	Name	Meaning
A0	angle_0_1_2	Neck angle (right)
A1	angle_0_1_5	Neck angle (left)
A2	angle_1_2_3	Right shoulder angle
A3	angle_1_5_6	left shoulder angle
A4	angle_2_3_4	Right elbow angle
A5	angle_5_6_7	Left elbow angle
A6	angle_12_9_10	Right hip angle
A7	angle_9_12_13	Left hip angle
A8	angle_9_10_11	Right knee angle
A9	angle_12_13_14	Left knee angle

The distance between joints has a similar significance to the angle between them In biological movement. An earlier study found that the left upper arm motion system is made up of the left shoulder joint and left elbow joint (25). The variations between the two joints could be described using the transverse and longitudinal distances between joints. As indicated in Table 2, we presented a total of 13 joint distance measures in our study, covering 26 critical elements. For example, D7, i.e., the distance between Nos. 4 and 7 symbolizes the human hand swing. D13, i.e., the distance between Nos. 11 and 14 can demonstrate the amplitude of foot stride. Particularly, leg swing denotes the length of the entire leg, whereas thigh swing concentrates on the distance from the hip to the knee. We considered both the length of the total leg and the distance between the hip and the knee in our analysis because they are both crucial gait characteristics (26).

**TABLE 2:** The meaning of distance between joints.

Number	Name	Meaning
D1 (0 and 1)	dist_1_0_x and dist_1_0_y	Head swing
D2 (2 and 3)	dist_3_2_x and dist_3_2_y	Upper arm swing (left)
D3 (4 and 5)	dist_4_2_x and dist_4_2_y	Arm swing (left)
D4 (6 and 7)	dist_6_5_x and dist_6_5_y	Upper arm swing (right)
D5 (8 and 9)	dist_7_5_x and dist_7_5_y	Arm swing (right)
D6 (10 and 11)	dist_6_3_x and dist_6_3_y	Arm elbow swing
D7 (12 and 13)	dist_7_4_x and dist_7_4_y	Swing with both hands
D8 (14 and 15)	dist_10_9_x and dist_10_9_y	Left thigh swing
D9 (16 and 17)	dist_11_9_x and dist_11_9_y	Left leg swing
D10 (18 and 19)	dist_13_12_x and dist_13_12_y	Right thigh swing
D11 (20 and 21)	dist_14_12_x and dist_14_12_y	Right leg swing
D12 (22 and 23)	dist_13_10_x and dist_13_10_y	Knee joint swing of both legs
D13 (24 and 25)	dist_14_11_x and dist_14_11_y	Foot stride

The original static data with unified frame number, inter-frame difference, inter-joint distance, and inter-joint angle were used to obtain the static and dynamic gait information. Ten time-domain feature extraction indicators, such as mean, variance, root means square, and others were employed (Table 3).

**TABLE 3:** The 10 time-domain feature extraction indicators used in the study.

Number	Characteristic function	Meaning
1	Maximum (x)	Calculating the maximum value of time series X
2	Minimum (x)	Calculating the minimum value of time series X
3	Mean (x)	Calculating the mean value of time series X
4	Median (x)	Returning the median of X
5	Variance (x)	Calculating the variance of time series X
6	Root_mean_square (x)	Calculating the root mean square of time series X
7	Skewness (x)	Calculating the sample skewness of X
8	Kurtosis (x)	Calculating the kurtosis of X
9	Abs_energy (x)	Calculating the absolute energy of time series X
10	Variation_coefficient (x)	The coefficient of variation of time series X is calculated

The features of four different types of data in the time domain information data pool were extracted using the aforementioned ten indexes. A total of 1,320 dimensional time-domain characteristics (1320 = 48 * 10 + 48 * 10 + 26 * 10 + 10 * 10) were obtained (Figure 3).

#### Frequency-domain features

Fourier transform is frequently employed in frequency domain analysis to observe a signal’s spectrum, but it is ineffective for studying signals whose frequency varies over time. The problem remains that the window function does not vary with frequency, even though the short-time Fourier transform (STFT) was built on this basis to provide time-frequency localization by adding a moving window function. A fixed window function is used by STFT. The form of the window function and the STFT’s resolution are both fixed once they are decided. The window function must be re-selected to modify the resolution. The above issues are fixed by the wavelet transform (WT), which also offers a “time-frequency” window that alters with frequency to enable multi-resolution analysis.

Different joints move at different amplitudes during gait; for instance, the limbs move at an amplitude that is noticeably higher than the trunk and head. Some crucial points in a particular gait sequence display a high-frequency trend, whereas others display a low-frequency trend. The wavelet transform was used in this work for gait time-frequency analysis based on the properties of the gait data and the wavelet transform’s function.

In this study, the gait time series data with uniform frame numbers were decomposed into a five-level wavelet decomposition using the “Haar” wavelet foundation. The low-frequency signal a was produced as A1 and the high-frequency signal as D1 following the first wavelet decomposition. The low-frequency signal received from the previous decomposition was then split into two parts: low frequency and high frequency, according to the decomposition results of each layer. The source signal x was decomposed into the following after five-level wavelet decomposition:


(2) $$X=D_{1}+D_{2}+D_{3}+D_{4}+D_{5}+A_{5}$$

**FIGURE 4:**
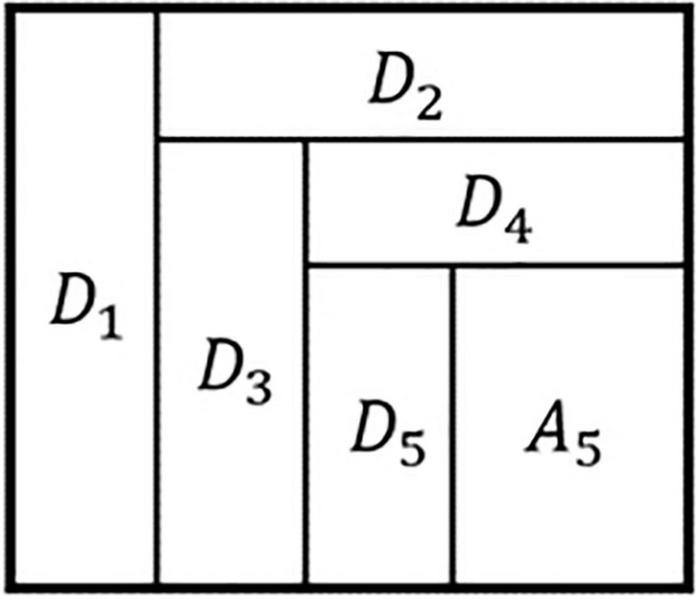
Schematic diagram of wavelet decomposition.

The decomposed high-frequency signals from Layers One through Five were designated as D1, D2, D3, D4, and D5 (also known as detail coefficients array), accordingly. According to Figure 4, the low-frequency signal A5 was produced by the fifth level decomposition (approximation coefficients array).

Absolute maximum, mean, variance and absolute energy were calculated separately for each detail coefficient array and approximate coefficient array to produce 1152 (1152 = 48 * 6 * 4) dimensional frequency domain features.

### Model training

After feature extraction, 2,472-dimensional gait time-frequency features were obtained. We employed sequential forward selection for feature selection. To train the machine learning models with 10-fold cross validation, we used a set of classifiers that included Gaussian processes (GP), linear regression (LR), Random Forest Regression (RFR), linear-support vector regression (linear-SVR), poly-support vector regression (poly-SVR), RBF-support vector regression (RBF-SVR), and sigmoid support vector regression (sigmoid-SVR). We chose those models since they were popular in predicting psychological traits and suitable for time series and imbalance data analysis (27, 28).

## Results

The mean score of the RESE was 31.40 (SD = 4.55). We collected about 3,000 frames of gait data that lasted nearly 2 min for every participant (25 * 60 * 2 = 3000).

**FIGURE 5:**
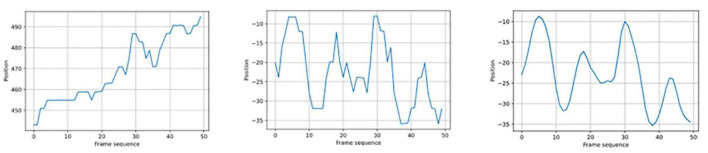
A comparison of the signal before and after the translation and Gaussian filter.

As the result of the Gaussian filter, we used the LWrist’s Y-axis data as an example here. The new Y-axis data (Figure 5 right) were smoother than the original Y-axis data (Figure 5 left) after translation in accordance with the MidHip (Figure 5 middle) and noise reduction using the Gaussian filter.

The correlation coefficients between the predicted self-esteem score and the self-report score as well as the mean squared error (MSE) were used to grade the models. MSE is a popular performance statistic in regression tasks. It denotes the degree of dispersion between the predicted and true values (29). Table 4 demonstrated that each correlation was significant. For all individuals, males, and females, respectively, GP, LR, and GP had the highest correlations of 0.51, 0.52, and 0.46. Simultaneously, GP had the lowest MSE of 4.08, followed by LR of 4.12.

**TABLE 4:** The mean squared error and the correlations between the model-predicted scores and the self-reported self-esteem scores.

Algorithm	MSE	*r*	*r*_*male*_	*r*_*female*_
GP	4.08	0.51***	0.44***	0.46***
LR	4.12	0.50***	0.52***	0.45***
RFR	4.30	0.30***	0.22***	0.29***
Linear-SVR	5.42	0.08**	0.12***	0.03
Poly-SVR	4.31	0.30***	0.20***	0.36***
RBF-SVR	4.17	0.42***	0.46***	0.36***
Sigmoid-SVR	4.15	0.35***	0.39***	0.28***

GP, Gaussian processes; LR, linear regression; RFR, Random Forest Regression; SLR, simple linear regression; ***p < 0.001, **p < 0.01.

### Reliability

We evaluated the split-half reliability of the models to further verify their effectiveness. Split-half reliability is the correlation between two parallel sub-tests, i.e., the odd sub-test and the even sub-test in our study. Specifically, to calculate the predicted values of the original model, we divided the 75 frames of gait data into odd and even half. Then, we computed the correlation coefficients of the two predicted values. In terms of reliability, RBF-SVR attained the highest value of 0.92 (Table 5).

**TABLE 5:** The odd-even split reliability of the model.

Algorithm	*r*
GP	0.79***
LR	0.83***
RFR	0.83***
Linear-SVR	0.76***
Poly-SVR	0.34***
RBF-SVR	0.92***
Sigmoid-SVR	0.87***

GP, Gaussian processes; LR, linear regression; RFR, Random Forest Regression; SLR, simple linear regression; ***p < 0.001.

### Feature weight

**FIGURE 6:**
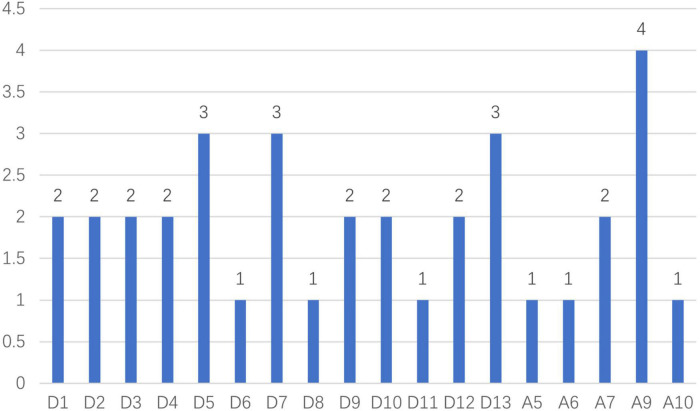
The role of each feature.

We also analyzed the weight of each component in the prediction process to determine which features contributed more to the outcome. After carefully evaluating the correlations and reliabilities of those classifiers, we settled on the linear regression because both of its outcomes were comparatively impressive. According to the results (Figure 6), A9, D5, D7, and D13 were more important in the prediction process (The result with a value of 0 was not shown in the figure). Additionally, when predicting self-esteem (*t* = 2.61, *df* = 21, *p* = 0.016), distances (*M* = 2.00, SD = 0.71) were more significant than angles (*M* = 0.90, SD = 1.29).

## Discussion

This study not only supports prior research that showed behavior markers might be used to assess self-esteem and demonstrates that gait patterns can reflect self-esteem rather effectively, but it also represents a considerable advance in comparison to earlier studies (14, 16). More significantly, the use of an omnipresent camera enables the implementation of a real-time, population-based prediction of human psychological traits (self-esteem) scores. Our study demonstrates that participants’ self-esteem may be accurately predicted after just 2°min of walking. To our knowledge, this is the first real-time study to quantify individual psychological traits on a large scale by using simple public-available facilities.

We collected 2D gait data from 152 participants using a ubiquitous camera, and then we extracted characteristics to create a machine learning model that may disclose their level of self-esteem. For all individuals, males, and females, respectively, GP, LR, and GP had the highest correlations of 0.51, 0.52, and 0.46. Moreover, RBF-SVR achieved the greatest dependability of 0.92. Moreover, this study has shown that A9, D5, D7, and D13 play greater roles in prediction. Additionally, distances were a better predictor of self-esteem than angles, which is consistent with earlier research that found no significant association between forward head posture angles and self-esteem, despite a negative medium link between round shoulder distance index and self-esteem (*r* = 0.35, *p* < 0.05) (30).

This study differs significantly from previous ones in that we focus on the dynamic shift in body posture rather than the static human position in each frame separately, which is better able to capture the essence of gait. The procedure is more appropriate because our analysis relies on 2D data rather than 3D data for its outcomes. More importantly, using feature selection with a theoretical foundation improves the explanation of the findings. Last but not least, we significantly expanded the sample size, which improved the reliability of the findings (16, 31).

The new method can be used to measure people’s self-esteem when they are in their familiar environment in addition to the benefits of behavior assessment mentioned in earlier studies, such as non-invasive and real-time because 2D cameras are now widely available. These benefits allow us to measure people’s self-esteem and obtain scores whenever and wherever we need to. For instance, it has been demonstrated that bullying or cyberbullying and low self-esteem are closely related (32). Bullying frequently happens on campuses, which has caused general concern in society. Using the campus cameras, we can regularly check on students’ self-esteem to identify the critical groups and assist in the prevention of negative events. Additionally, this approach might be used during interviews because the way respondents complete questionnaires will be influenced by social acceptance, which will lead to unreliable answers. This technique not only improves the ecological validity of self-esteem measurement and offers a substitute for individuals who cannot read or write, but it also shifts the paradigm of psychological research to make use of common public-available resources.

Of course, this study has certain limitations. The first is that we only tested the models using the RSES. Future studies could incorporate more criteria. Additionally, every participant is a graduate student. Large sample populations with a variety of jobs, ages, and cultural groups could be used in future studies to advance the generation of the result.

This study provides an innovative and practical method to measure self-esteem. The tool used to capture gait data expands the previous research which makes the data collection more convenient and considerably expands the application scenarios, and the results confirm the validation of behavior assessment for self-esteem. This method can be a useful supplementary method to the existing self-esteem measurements.

## Data availability statement

The raw data supporting the conclusions of this article will be made available by the authors, without undue reservation.

## Ethics statement

The studies involving human participants were reviewed and approved by the Institutional Review Board of the Institute of Psychology at the Chinese Academy of Sciences. The patients/participants provided their written informed consent to participate in this study.

## Author contributions

TZ and XL developed the idea, methodology, and provided supervision. YW contributed to method implementation and analysis. TZ was involved in project administration. All authors contributed to the manuscript, read, and approved the submitted version.
